# Short-term outcomes of acute coronary involvement in type A aortic dissection without myocardial ischemia: a multiple center retrospective cohort study

**DOI:** 10.1186/s13019-021-01469-z

**Published:** 2021-04-23

**Authors:** Maozhou Wang, Ruixin Fan, Tianxiang Gu, Chengwei Zou, Zonggang Zhang, Zhonghong Liu, Chenhui Qiao, Lizhong Sun, Ming Gong, Haiyang Li, Hongjia Zhang

**Affiliations:** 1 Department of Cardiac Surgery, Beijing Chaoyang Hospital, Capital Medical University, 8 South Road of Workers Stadium, Chaoyang District, Beijing, China; 2 Present address: Beijing Anzhen Hospital, Capital Medical University, Beijing, China; 3 Guangdong General Hospital, Guangzhou, Guangdong China; 4 China Medical University First Hospital, China Medical University, Shenyang, Liaoning China; 5 Shandong Provincial Hospital, Jinan, Shandong China; 6 People’s Hospital of Xinjiang Uygur Autonomous Region, Urumqi, Xinjiang, Uygur Autonomous Region China; 7 First Affiliated Hospital of Harbin Medical University, Harbin Medical University, Harbin, Heilongjiang China; 8 Zhengzhou University First Affiliated Hospital, Zhengzhou University, Zhengzhou, Henan China; 9 Department of Cardiac Surgery, Beijing Anzhen Hospital, Capital Medical University, 2 Anzhen Road, Beijing, Chaoyang District China

**Keywords:** Acute type a aortic dissection, Acute coronary involvement, Without myocardial ischemia, Short-term outcomes

## Abstract

**Background:**

To evaluate the early prognosis and management of acute coronary involvement (ACI) in type A aortic dissection (ATAAD) patients without myocardial ischemia (MI).

**Methods:**

We conducted a retrospective cohort study on a multicenter database. A total of 931 ATAAD patients without MI underwent thoracic aortic surgery between 2018 and 2019 in the Acute Aortic Syndrome Cooperation Network (AASCN) and were enrolled in our study. Patients were divided into two groups: ACI group and non-ACI group.

**Results:**

There were 139 ACI patients (14.9%) and 792 non-ACI patients (85.1%) in our cohort. ACI group had higher 30-day mortality after surgery than non-ACI group (log-rank test: *P* = 0.028,Cox regression: hazard ratio [HR], 2.3; 95% confidence interval [95% CI], 1.1–5.39; *P* = 0.047), especially in sub-group of advanced age (53–80 years; HR, 4.0; 95% CI, 1.3–12.8; *P* = 0.017), low diastolic blood pressure (29-69 mmHg, HR, 3.8; 95% CI, 1.3–11.2; *P* = 0.018), low systolic blood pressure (51–119 mmHg, HR, 3.6; 95% CI, 1.1–12.4; *P* = 0.040), high body mass index (BMI;27.25–47.52 kg/m^2^; HR, 3.7; 95% CI, 1.3–10.7; *P* = 0.015) and high hemoglobin (>145 g/L; HR, 4.3; 95% CI, 1.2–16.0; *P* = 0.030). Acute renal failure was significant more in ACI group than non-ACI group (24.5% vs. 15.9%; *P* = 0.014).

**Conclusions:**

ACI increases the short-term postoperative mortality and acute renal failure in ATAAD patients without MI. ATAAD patients with ACI may need a narrower control range of blood pressure even if without myocardial ischemia.

**Trial registration:**

ChiCTR1900022637. Retrospectively registered 19 April 2019.

## Introduction

Acute aortic dissection is a serious condition caused by the rupture of the aortic intima, causing blood to enter the aortic wall through the tear [1]. Acute type A aortic dissection (ATAAD) characteristically poses a high risk of death and multiple complications that need effective management [2, 3]. One such complication is acute coronary involvement (ACI), with a rate of 7–15% [4–6].

ACI refers to the tear of proximal aortic dissection to coronary artery. Myocardial ischemia (MI) resulting from ACI leads to poor outcomes due to postoperative heart failure or acute myocardial infarction [7, 8], but not all ACI patients suffer from MI upon admission. Some ACI patients have no signs of MI and are diagnosed by computed tomography, transesophageal echocardiography or intraoperative exploration [9]. However, the statistical evidence and guidelines of the ACI in ATAAD patients without MI are limited. Besides, there are no report about the management of ACI in ATAAD patients without MI. Treatment options mainly depend on the heart surgeon’s individual experience.

Based on the Acute Aortic Syndrome Cooperation Network (AASCN) of China, we aim to determine the effect of ACI and different management on early postoperative mortality in ATAAD without MI.

## Patients and methods

### Study population

The AASCN contains the database of patients who suffered from acute aortic syndrome at ten heart centers (Beijing Anzhen hospitcal; Beijing Chaoyang Hospital; Guangdong General Hospital; China Medical University First Hospital; Shandong Provincial Hospital; People’s Hospital of Xinjiang Uygur Autonomous Region; First Affiliated Hospital of Harbin Medical University; Zhengzhou University First Affiliated Hospital; Ruijin Hospital and Sir Run Run Shaw Hospital) in China from April 2018, to December, 2019. The follow-up period was 10 years. Hospital related data were collected by a third party within 1 week after discharge. The long-term follow-up was mainly by telephone. The database is constantly updated. This study is mainly led by Anzhen Hospital, Beijing, China, and was approved by the hospital’s Ethics Committee in April 2018 (No. 2018004). The need for informed consent was waived.

### Study cohort

We used ACI with ATAAD as an exposure factor to assemble a retrospective cohort study from the AASCN and eliminated patients without ATAAD, with MI or lack of follow-up. Missing data: there were 42 patients without record of ACI type, 39 patients without record of coronary artery treatment, 15 patients without indicators of myocardial ischemia before operation, 9 patients without record of operation type, 25 patients without the record of preoperative blood pressure, 4 patients without the record of cardiopulmonary bypass time, 8 patients without the record of circulatory arrest time. Ultimately, 931 ATAAD patients without MI were enrolled in our study. We observed patients from admission to 30-days postoperatively or death earlier than 30-days postoperatively. Diagnosis of ACI was mainly based on preoperative aortic computed tomography angiography (CTA) (after onset) with coincident of at least one cardiac surgeon and one radiologist or some suspicious ACI (according to CTA) were diagnosed based on direct vision of the cardiac surgeon intraoperatively (Tear of coronary artery ostium under direct vision) [ 4, 6].According to the CTA and Neri’s classification of ACI [10], we classified them into 3 types: type A (aortic dissection with a false lumen that involves the coronary ostium without coronary actual body); type B (aortic dissection false lumen that extends into the actual body of the coronary with successful administration of antegrade cardioplegia) and type C (crack of coronary artery). We divided these patients into ACI group and non-ACI group.

### Definitions and end-point

We defined MI as elevated sensitive troponin I value with at least 1 value above the 99th percentile upper reference limit and combine one of the following: electrocardiogram indicates ST segment depression or elevation and abnormal wall motion indicated by echocardiography [11]. The type of ACI refers to Neri’s classification [10]. The primary end-point was all-cause death within 30 days after surgery. The secondary end-point were new-onset acute renal failure, low cardiac output syndrome, and malignant arrhythmia within 30 days after operation. Low cardiac output syndrome was defined as a cardiac index of < 2.2 L/min/m^2^ in the absence of hypovolemia [central venous pressure ≥ 8 mmHg and/or pulmonary capillary wedge pressure ≥ 12 mmHg and/or diastolic pulmonary artery pressure ≥ 12 mmHg] [12]. Acute kidney was defined according to KDIGO guidelines [13]. Malignant arrhythmia was defined as ventricular arrhythmias that cause hemodynamic changes [14].

### Surgical techniques

All surgeries were performed by experienced cardiac surgeons (>30 aortic dissection surgery per year and postoperative mortality less than 10%). All ATAAD patients without surgical contraindications received surgery at the first time [15, 16]. All centers followed the same principles of major vascular surgery committee of cardiovascular surgery branch of Chinese Medical Association [16]. Cardiopulmonary bypass was performed in all patients in our study. Deep-hypothermia circulatory arrest and selective cerebral perfusion were performed if the aortic dissection involves the brachiocephalic vessels.

#### Distal and proximal aortic repair

All patients received surgery according to the previously described standardized procedure [15, 16]. For the proximal aortic repair, the ascending aortic replacement was performed in patients with ascending aortic dissection (AAD) without any other problem (aortic valve or aortic sinus problem). Bentall (a valve combined aortic root replacement) was performed in AAD patients with aortic valve and aortic sinus problem. David IV was performed in AAD patients with valve problem without aortic sinus problem.

Distal repair included the aortic arch repair, total arch replacement and hemiarch replacement. Aortic arch repair was performed in patients with uninvolved brachiocephalic vessels. Total arch replacement was performed in patients with total brachiocephalic vessels involved. Hemiarch replacement was performed in patients with part of brachiocephalic vessels involved.

#### Coronary ostium repair (COR) and coronary artery bypass grafting (CABG)

COR or CABG were performed according to Neri’s classification [10]. We consider that it is very important to explore the involvement of coronary artery ostium carefully for these patients without myocardial ischemia. Generally, the final type of ACI can be determined during the operation. For type A (Neri’s classification) patients, coronary ostium repair (COR) is the first choice. However, if the effect of COR is not good in some patients with type A (presented with coronary ostial stenosis after COR), we recommend switch to coronary artery bypass grafting (CABG). For type B patients, because the stenosis of coronary is not clear (whether CTA or intraoperative exploration may not fully determine the severity of coronary stenosis), we recommend CABG should be considered more actively to ensure coronary blood supply. For type C, the first choice is CABG, but there is no type C patients in our cohort. In addition, due to our study is a multicenter retrospective cohort analysis from database, there may be differences in the specific methods of COR and CABG from each center. According to the experience of our center (Beijing Anzhen Hospital), the specific method of COR are as follows: Start suturing from the junction of the left and right coronary sinus to the junction of the right or left coronary sinus and the noncoronary sinus by 6–0 prolene (Artificial shim are required for both the first needle and the last needle, suture along the upper edge of the affected coronary artery). When CABG was needed, considering the longer time of internal mammary artery acquisition, the great saphenous vein graft (SVG) was the first choice in all these patients. SVG was harvested and anastomosed to the distal of the involvement coronary in a standard manner with 8–0 prolene. Closing the affected coronary ostium with 5–0 prolene and then cardioplegia was applied through the proximal SVG. Proximal SVG was anastomosed to the brachiocephalic vessels after cardiac resuscitation.

### Statistical analysis

We used PASS 15 to calculate the sample size (two-sided, power:0.90 Alpha:0.05, Hazard ratio is 0.5, duration time is 1 day to 30 day), the numeric results for the log-rank test in terms of sample size were 48 in non-ACI group and 49 in ACI group. Missing data were deleted (*n* = 146, 11.7%). Continuous variables conforming to normal distribution with homoscedasticity were analyzed via independent-sample t test and expressed as a mean with a standard deviation (SD). Continuous variables that do not conform to normal distribution were analyzed via Wilcoxon rank sum test and expressed as an interquartile range (IQR). Categorical variables were presented as frequencies with percentages and analyzed by chi-square or Fisher’s exact test, as appropriate. Kaplan-Meier plot was used to estimate survival rate and log-rank test was applied to intergroup comparisons. Risk factors for 30-day mortality were identified using cox regression. The variables in univariable factor analysis with *P<*0.1 or risk factors previously reported were included in Cox regression model [17–20]. Stratified analysis was used to identify differences of 30-day mortality among subgroups. In stratified analysis, continuous variables were categorized equally into three groups according to the number of people. Category boundaries were determined according to the separation value after equal division. Hazard Ratio (HR) was used to represents the difference of log-rank test between the two groups. We set the confidence interval to 95% (95%CI) in survival analysis. A two-tailed *P* < 0.05 indicated statistical significance. We used R software version 3.4.3 (Ihaka and Gentleman, 1996) for all of the above analysis.

## Results

### Baseline characteristics

**Fig. 1:**
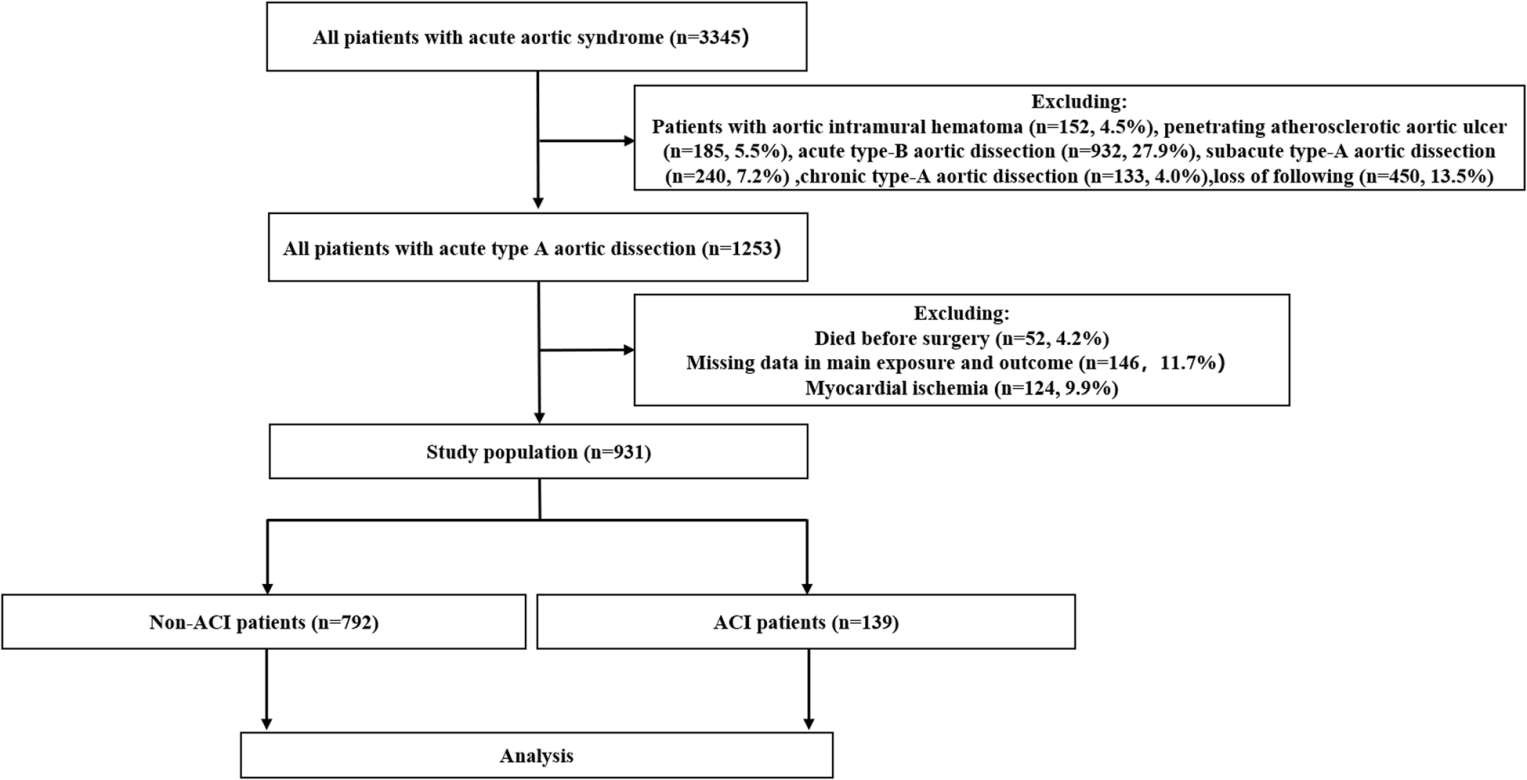
Selection process

We ultimately enrolled 931 patients in our cohort; 139 were ACI and 792 were non-ACI. The selection process was presented in Fig. 1.

Baseline characteristics were summarized in Table 1. In terms of general information, the ACI group had higher BMI (*P* = 0.005). In terms of examination and experimental results, there were no significant difference between two groups.

**Table 1:** Baseline of ATAAD without MI^a^

Variable	ACI group	non-ACI group	*P*-value
	(*n* = 139)	(*n* = 792)	
General information			
Age, mean (SD)	48.4 (9.3)	49.0 (10.0)	0.546
Male, n (%)	110 (79.1)	649 (81.9)	0.431
BMI^b^, mean (SD) (Kg/M^2^)	26.9 (3.8)	25.9 (3.9)	0.005^***^
Heart rate, mean (SD)	81.6 (11.6)	81.9 (13.1)	0.827
SBP^c^, mean (SD) (mmHg)	128.7 (17.6)	132.3 (23.0)	0.087
DBP^d^, mean (SD) (mmHg)	74.6 (13.3)	75.5 (15.0)	0.511
Laboratory examination			
EF, mean (SD) (%)	61.1 (6.4)	58.1 (6.9)	0.078
Platelet, mean (SD) (10^9^/L)	171.3 (56.7)	182.1 (74.3)	0.104
hemoglobin, mean (SD) (g/L)	135.4 (19.6)	133.3 (26.4)	0.355
D-dimer, median (IQR) (ng/ml)	2365 (5811.5)	2297 (6160)	0.324
Previous history			
Marfan, n (%)	2 (1.4)	18 (2.3)	0.755
History of previous heart surgery, n (%)	5 (3.6)	24 (3.0)	0.790
Hypertension, n (%)	108 (77.7)	579 (73.1)	0.256
Aortic insufficiency (2+), n (%)	41 (29.5)	222 (28.0)	0.723
Coronary heart disease, n (%)	5 (3.6)	45 (5.7)	0.415
Diabetes, n (%)	4 (2.9)	26 (3.3)	1.000
Stroke, n (%)	2 (1.4)	33 (4.2)	0.148
Pericardial effusion, n (%)	29 (20.9)	179 (22.6)	0.650

^a^Baseline of acute type A aortic dissection patients without myocardial ischemia between acute coronary involvement and no acute coronary involvement; ^b^Body mass index; ^c^Systolic blood pressure of first arrive at the emergency; ^d^Diastolic blood pressure of first arrive at the emergency.^***^: *P<*0.001

### Operative and postoperative outcomes

Table 2 showed operative and postoperative outcomes of ACI group and non-ACI group. In terms of surgical methods, ACI patients received more CABG than non-ACI patients (21.6% vs. 4.3%; *P* < 0.001) and the ACI group had longer duration of surgery (476.3 min vs. 447.6 min; *P* = 0.004), longer duration of cardiopulmonary bypass (231.6 min vs. 203.4 min; *P* < 0.001).

**Table 2:** Operative and postoperative outcomes

Variables	ACI group^a^	non-ACI group^b^	*P*-value
	n = 139	n = 792	
Proximal repair			
Bentall^c^, n(%)	51 (36.7)	261 (33.0)	0.389
David^d^, n(%)	1 (0.7)	11 (1.4)	1.000
Wheat^e^, n(%)	1 (0.7)	2 (0.2)	0.385
AAR^f^, n(%)	86 (61.9)	518 (65.4)	0.421
Distal repair, n(%)			
Aortic arch formation, n(%)	2 (1.4)	11 (1.4)	1.000
Hemiarch replacement, n(%)	12 (8.6)	66 (8.3)	0.906
Total arch replacement, n(%)	101 (72.7)	602 (76.0)	0.397
ACI side^g^			
Left coronary artery, n(%)	12 (8.6)	–	–
Right cornary artery, n(%)	129 (92.8)	–	–
Bilateral coronary artery, n(%)	2 (2.3)	–	–
ACI classification^h^			
Type A, n(%)	113 (81.3)	–	–
Type B, n(%)	16 (18.7)	–	–
Type C, n(%)	0 (0)	–	–
Manamement of ACI			
COR^i^	109 (78.4)	–	–
CABG^j^	30 (21.6)	37 (4.7)	< 0.001^***^
Duration of surgery, mean (SD),min	476.3 (127.2)	447.6 (115.5)	0.008^**^
Duration of cardiopulmonary bypass, mean (SD),min	231.6 (74.6)	203.4 (60.7)	< 0.001^***^
Duration of ICU^k^, mean (SD), hour	134.6 (211.2)	176.5 (487.5)	0.320
Duration of MV^l^, mean (SD), hour	82.1 (125.1)	80.6 (110.2)	0.888
Secondary outcomes			
LCOS^m^, n(%)	8 (5.8)	28 (3.5)	0.211
Acute kidney injury, n(%)	34 (24.5)	126 (15.9)	0.014^*^
Malignant arrhythmia, n(%)	1 (0.7)	13 (1.6)	0.410

^a^Acute coronary involvement in acute type A aortic dissection patients without coronary malperfusion; ^b^No acute coroanry involvement in acute type A aortic dissection patients without coroanry malperfusion; ^c^Bentall procedure: total ascending aortic, aortic sinus and aortic valve replacement; ^d^David procedure: total ascending aortic and aortic sinus replacement with preservation of aortic valve; ^e^Wheat:total ascending aortic and aortic valve replacement with preservation of aortic sinus; ^f^AAR procedure:simple total ascending aortic relacement; ^g^The side of acute coronary involvement; ^h^Acute coronary involvement anatomical classification according to Neri et al.; ^i^Coronary ostium repair; ^j^Coronary artery bypass grafting; ^k^Duration of staying in intensive care unit;^l^Duration of mechanical ventilation; ^m^: low cardiac output syndrome ^***^: *P<*0.001;^**^: *P<*0.01,^*^:*P<*0.05

### Primary outcomes

**Fig. 2:**
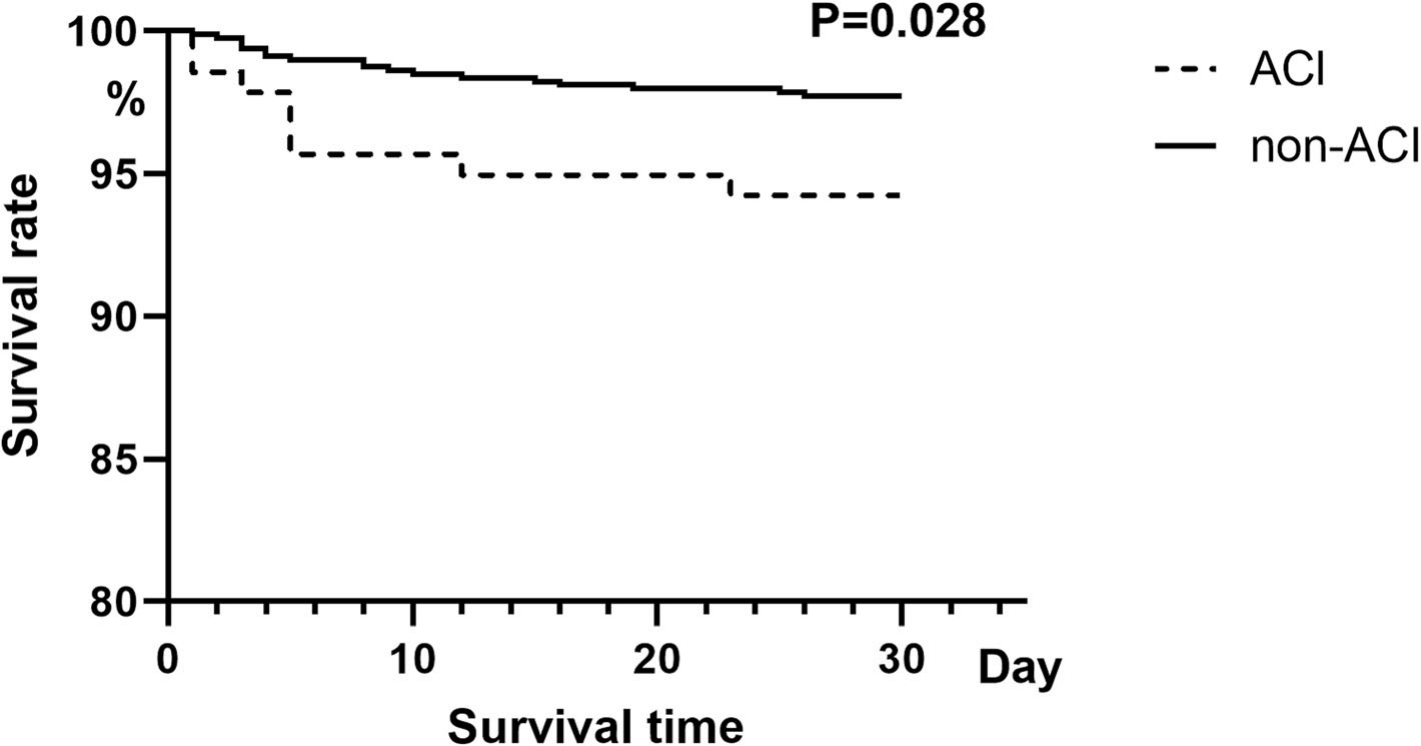
Kaplan-Meier curves of ACI and non-ACI in ATAAD without MI

**Fig. 3:**
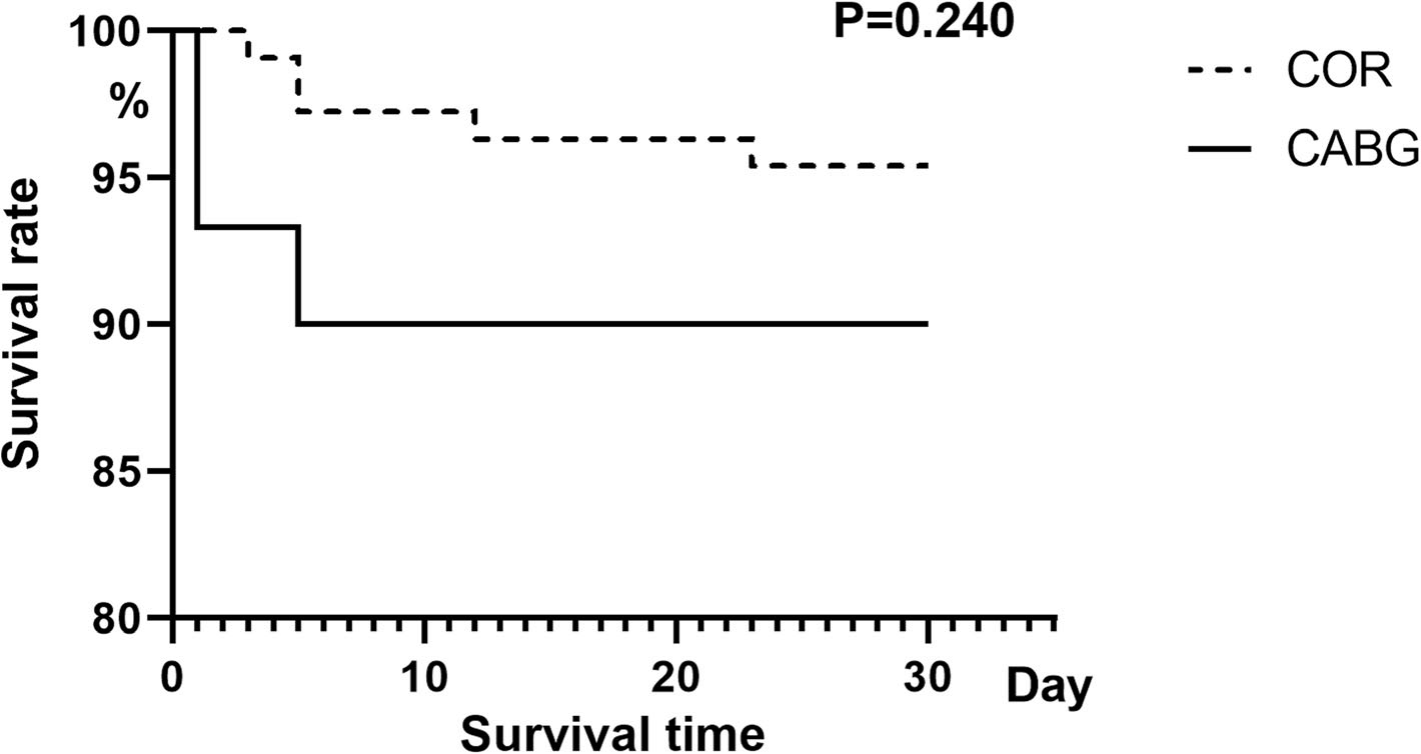
Kaplan-Meier curves of COR and CABG in ATAAD with ACI but without MI

Hospital mortality within 30 days was 8/139 (5.8%) in ACI group and 10/792 (2.4%) in non-ACI group. The absolute risk for early death of ACI is 3.4%. As shown in Fig. 2, the ACI group had shorter postoperative survival rate in 30 days (28 days vs. 29 days, log-rank test, *P* = 0.028). In the multiple Cox regression model, as shown in Table 3, independent risk factors were BMI (hazard ratio [HR], 1.13; 95% confidence interval [CI], 1.03–1.25; *P* = 0.012), CAD (HR, 3.13; 95% CI, 1.04–9.40; *P* = 0.042) and ACI (HR, 2.34; 95% CI, 1.01–5.39; *P* = 0.047). The 30-day mortality was no significant difference between CABG and COR (log-rank test *P* = 0.240) (Fig. 3).

**Table 3:** Cox regression of 30 days mortality in ATAAD without MI^a^

Variables	HR	95% CI	*P*-value
Gender	1.762	0.724–4.288	0.212
Age	1.024	0.982–1.070	0.264
BMI^b^	1.134	1.028–1.250	0.012^*^
History of previous heart surgery	0.899	0.119–6.815	0.918
EF^c^	0.985	0.934–1.038	0.568
Coronary artery disease	3.128	1.0140–9.404	0.042^*^
ACI^d^	2.336	1.013–5.386	0.047^*^
Stroke	0.971	0.130–7.282	0.978

^a^*ATAAD* Acute type A aortic dissection, *MI* Myocardial ischemia; ^b^Body mass index;^c^Ejection Fraction; ^d^Acute coronary involvement; ^*^: *P<*0.05

### Secondary outcomes

As shown in Table 2, ACI group had more postoperative renal failure compared to non-ACI group (24.5% vs 15.9%; *P* = 0.014). There was no significant difference in low cardiac output syndrome and malignant arrhythmia between two groups.

### Stratified analysis and interaction test for primary outcomes

As shown in Table 4, ACI were related to higher 30-day postoperative mortality than non-ACI group in sub-group of advanced age (53–80 years; HR, 4.0; 95% CI, 1.3–12.8; *P* = 0.017), low diastolic blood pressure (29-79 mmHg, HR, 4.3; 95% CI, 1.7–11.4; *P* = 0.003), low systolic blood pressure (51–119 mmHg, HR, 3.6; 95% CI, 1.1–12.4; *P* = 0.040), high body mass index (BMI; HR, 3.7; 95% CI, 1.3–10.7; *P* = 0.015) and high hemoglobin (>145 g/L; HR, 4.3; 95% CI, 1.2–16.0; *P* = 0.030). None of the confounding factors interacted with ACI in 30-day postoperative mortality (interaction *P* > 0.05).

**Table 4:** Stratified analysis and forest plot of ACI on short-term mortality in ATAAD without MI^a^

Confounding factors	Stratification	n(%)	HR	95%CI	P	Interaction P
Age, years	18–46	308 (33.1)	1.1	0.1–9.6	0.918	0.474
	47–52	311 (33.4)	1.8	0.4–8.5	0.476	
	53–80	312 (33.5)	4.0	1.3–12.8	0.017^*^	
DBP^b^, mmHg	29–69	249 (26.7)	3.8	1.3–11.2	0.018^*^	0.576
	70–79	282 (30.3)	2.7	0.2–29.4	0.423	
	80–164	400 (43.0)	1.4	0.3–6.6	0.664	
SBP^c^, mmHg	51–119	231 (24.8)	3.6	1.1–12.4	0.040^*^	0.702
	120–139	364 (39.1)	1.6	0.3–7.8	0.578	
	140–216	336 (36.1)	2.3	0.5–11.6	0.296	
BMI^d^, kg/m^2^	16.44–24.24	310 (33.3)	1.4	0.2–11.0	0.776	0.484
	24.34–27.24	241 (25.9)	1.2	0.1–10.3	0.899	
	27.25–41.52	380 (40.8)	3.7	1.2–10.3	0.015^*^	
Hemoglobin,g/L	26–120	220 (23.6)	0.9	0.1–7.1	0.879	0.561
	121–135	245 (26.3)	1.8	0.2–16.5	0.584	
	136–145	222 (23.8)	3.1	0.5–18.8	0.210	
	145–238	244 (26.2)	4.3	1.2–16.0	0.030^*^	

^a^:*ACI* Acute coronary involvement, *ATAAD* Acute type A aortic dissection, *MI* Myocardial ischemia; ^b^Diastolic blood pressure;^c^ Systolic blood pressure; ^d^Body mass index; ^*^: *P<*0.05

## Discussion

It has been reported that MI secondary to ATAAD increased early postoperative mortality, although ACI may occur without MI [6, 7]. The effect and management of ACI without MI were unclear. In our study, there were 124 ACI patents (9.9%) with MI and 139 ACI patients (11.1%) without MI. Our study indicated that ACI in ATAAD without MI was also related to higher early postoperative mortality than non-ACI. It suggests that ACI without MI should also be considered as an important preoperative risk factor.

In our study, the incidence of ACI in ATAAD was 20.7%, which was similar to that in another report [9]. BMI was not balanced in our baseline between ACI and non-ACI, so we performed Cox regression and stratified analysis to reduce and clarify the impact of this confounding factor. Cox regression showed that both ACI and BMI were the independent risk factors for early death. Stratified analysis showed that the ACI had a greater impact on early mortality in high BMI sub-group which indicated the independent superposition effect of ACI and BMI.

The reason of higher early mortality and postoperative acute renal failure of ACI than non-ACI in ATAAD without MI were summarized as follows:

1. Longer duration of surgery and cardiopulmonary bypass were needed for COR or CABG which would increase organ ischemia time especially for kidney [21]. Besides, some researchers reported that longer duration of cardiopulmonary bypass could be one of the reasons of in-hospital major adverse outcomes after surgery had in ATAAD [22].
2. In preparative time for surgery, some of ACI patients without MI progressed to suspected MI (Electrocardiogram showed ST change or esophageal ultrasound showed abnormal wall motion without sensitive troponin I change). Some ACI patients without MI may progress to ACI with MI owing to hemodynamic changes before surgery. As shown in our stratified analysis, ACI had a greater impact on early mortality in the subgroup of low systolic and diastolic blood pressure. Low blood pressure may lead to coronary malperfusion especially in ACI patients. Our study indicated patients with ACI may need a narrower control range of blood pressure in ATAAD even if without MI.

Older patients were more susceptible to ACI and had higher early postoperative mortality in our study. Some researchers reported that severe coronary atherosclerosis was more common in these patients [23]. More-serious coronary artery atherosclerosis might be one reason for more susceptible to ACI in this sub-group.

For the management of ACI, some researchers reported that preoperative percutaneous coronary intervention can increase the postoperative survival rate of ACI [ 24]. However, there was no big data evidence. Timely surgical treatment is still the first choice for these patients [15]. For the choice of coronary management, most cardiac surgeons decide mainly according to the Neri’s anatomic classification, but it is still controversial in the management (CABG or COR) of type A and type B of Neri’s classification in some cases [25]. All patients were suffered from type A or type B of Neri’s classification in our study. Our study showed that there was no significant difference between CABG and COR in early mortality of ACI in ATAAD patients without MI. However, early postoperative antiplatelet (<3 days after surgery) is important for the long-term patency of SVG after CABG [26]. it is contradictory between early antiplatelet after CABG and prevention of severe postoperative bleeding after ATAAD surgery. Therefore, COR might be the first choice for ACI in ATAAD patients without MI for better long-term outcomes.

Our study found that the early mortality of acute type A aortic dissection patients with acute coronary involvement (ACI) without myocardial ischemia was still high, especially in whom with low preoperative blood pressure. This study can provide the following reference for smaller centers and/or surgeons with lower annual volume:1, For patients with ATAAD, suspicious coronary artery problems from CTA should be paid more attention to. The absence of myocardial ischemia does not mean that myocardial ischemia will not occur. During the operation, we should develop the habit of exploring the bilateral coronary ostium. 2, Whether suspected or confirmed ACI by CTA, operation should be performed as soon as possible on the basis of maintaining a certain blood pressure (ensuring coronary perfusion), because of the progress of dissection, patients may develop myocardial ischemia. 3, The learning curve of aortic dissection surgery is long and experienced surgeons have better outcomes [27], so we should at least master the operation skills of aortic root surgery before trying. For patients with complex complications such as ACI, and for patients suspected of poor COR, CABG should be actively performed. According to our results, there was no significant difference between COR and CABG in the early mortality rate. Although we speculated that COR alone would be better for long-term outcomes, it is more important to ensure early postoperative survival.

Our study had some limitations. Firstly, our study is a retrospective cohort study based on the analysis of previous database data. We did not include patients with ACI combined myocardial ischemia in our study, and there may have some selection bias, but the sample size is enough, which has the generalizability. Secondly, it presented only early postoperative outcomes. We are still following up on middle and late postoperative outcomes of these patients. We consider that the long-term prognosis of these patients largely depends on the method of handling coronary ostium (coronary artery bypass grafting, CABG or coronary ostium repair, COR). Our hypothesis is that the long-term prognosis of COR patients is better than that of CABG patients. On the one hand, in order to ensure the true and false lumen closure, sometimes we should close the seriously affected coronary artery after CABG, and the affected coronary artery is completely supplied by the bypass vessel. Most CABG patients were only received great saphenous vein bypass in our study, and the long-term cardiovascular adverse events of great saphenous vein is about 47% with 10 years follow-up [28]. On the other hand, patients (without valve replacement) with CABG need to take dual antiplatelet which is a risk factor of bleeding related diseases and have more drug cost [29]. Secondly, our patient data came from heart centers in different regions of China. Because of the small number of ACI patients, we did not analyze them by region, which might cause offset.

## Conclusion

ACI increases the short-term postoperative mortality and acute renal failure in ATAAD patients without MI. ATAAD patients with ACI but without MI may need a narrower control range of blood pressure.

## Data Availability

The data that support the findings of this study are available from Acute Aortic Syndrome Cooperation Network (AASCN, http://118.26.69.165:23023/AASCN/index.html) but restrictions apply to the availability of these data, which were used under license for the current study, and so are not publicly available. Data are however available from the authors upon reasonable request and with permission of AASCN.

## Abbreviations

ACI: Acute coronary involvement
MI: Myocardial ischemia
ATAAD: Acute type A aortic dissection
AASCN: Acute Aortic Syndrome Cooperation Network
HR: Hazard ratio
DBP: Diastolic blood pressure
SBP: Systolic blood pressure
BMI: Body mass index
AAD: Ascending aortic dissection
COR: Coronary ostium repair
CABG: Coronary artery bypass grafting
SD: Standard deviation
SVG: The great saphenous vein graft
IQR: Interquartile range
